# EUROlinkCAT: A linked European cohort of children with congenital anomalies

**DOI:** 10.23889/ijpds.v8i2.2346

**Published:** 2023-09-14

**Authors:** Maria Loane, Joan Morris, Ester Garne, EUROlinkCAT Working Group

**Affiliations:** 1 Ulster University, Belfast, United Kingdom; 2 St George's University, London, United Kingdom; 3 Hospital Lillebaelt, Odense, Denmark

## Objectives

To establish a linked European cohort of children with congenital anomalies (CAs) to evaluate mortality and morbidity outcomes of these children up to the age of 10 years.

## Methods

EUROlinkCAT supported 22 EUROCAT population-based congenital anomaly registries in 14 countries to link their data on children with CAs to mortality, vital statistics, hospital discharge and prescription databases. All live births with a CA born 1995-2014 recorded in the registries were followed up to age 10 years or to 31st December 2015. Each registry transformed their local mortality and morbidity data to a Common Data Model (CDM) and ran centrally created syntax scripts and produced tables/outputs in a standard form for meta-analysis. Analyses were performed on 100 different congenital anomaly subgroups for children >1 year,1-4 years, and 5-9 years.

## Results

Sixteen registries linked their data on children with CAs to mortality databases, eleven to regional/national hospital databases, and six to prescription databases. Data on children without a CA born during the same time-period and from the same population area (reference population) were available for seven registries linking to hospital databases and for all six registries linking to prescription databases. For the mortality studies, linked information on survival was available for 96% of children recorded in the anomaly registries (180,00 live births). For the morbidity studies, 89% of children with a CA (n=99,000) and 95% of reference children (n=2 million) were linked. For the prescription studies, 95% of children with a CA (n=60,000) and 95% of reference children (n= 1,700,000) were linked.

## Conclusion

The EUROlinkCAT project was successful in creating a linked cohort of children with and without CAs in Western Europe. More efforts are needed to support data linkage in Eastern European countries. We have developed a set of recommendations for data linkage studies based on our experiences in establishing this cohort.

